# Brightening the path to safe liver transplants: the role of ICG fluorescence in biliary anastomosis

**DOI:** 10.1136/bmjsit-2024-000322

**Published:** 2025-06-09

**Authors:** Martín Huerta, Mar Dalmau, Nair Fernandes, Cristina Dopazo, Mireia Caralt, Laura Vidal, Ramón Charco, Itxarone Bilbao, Concepción Gómez-Gavara

**Affiliations:** 1 HPB Surgery and Liver Transplant Department, Vall d’Hebron University Hospital, Barcelona, Cataluña, Spain; 2 Autonoma University, Barcelona, Spain

**Keywords:** Health Technology, Technology

## Abstract

**Objectives:**

Evaluate the effectiveness of indocyanine green (ICG) fluorescence in enhancing the safety and precision of biliary anastomosis during liver transplantation (LT). The primary research question was whether ICG could provide real-time, objective assessment of bile duct vascularization to reduce postoperative biliary complications.

**Design:**

Prospective, observational case series. IDEAL Stage 1 study.

**Setting:**

Tertiary care academic medical center in Barcelona, Spain.

**Participants:**

10 adult patients who underwent LT between January 2023 and July 2024. Patients were selected based on the indication for LT with varying etiologies of liver failure. Donors included those with brain death and circulatory death (DCD).

**Interventions:**

ICG was administered intravenously as a 3 mg bolus dose to evaluate the vascularization of the bile duct stumps. Fluorescence was visualized using a high-definition camera system during surgery, and adjustments to the anastomosis site were made based on the fluorescence patterns observed.

**Main outcome measures:**

The primary outcome was the identification of non-vascularized (non-fluorescent) bile duct tissue and subsequent adjustments to the anastomosis site. Secondary outcomes included the incidence of biliary complications and patient survival during the follow-up period.

**Results:**

ICG fluorescence successfully identified non-fluorescent areas in the bile duct stumps, leading to surgical adjustments in five cases (50%), particularly in DCD grafts. The procedure was well-tolerated with no adverse events related to ICG administration. The use of ICG fluorescence added an average of 3–5 min to the operative time. No biliary complications were reported during follow-up, and patient survival was 100%.

**Conclusions:**

ICG fluorescence provides a valuable, objective tool for assessing bile duct vascularization during LT, potentially reducing biliary complications. This technique’s integration into clinical practice could enhance surgical precision and improve patient outcomes. Further research is needed to confirm these findings in larger, more diverse populations.

**WHAT IS ALREADY KNOWN ON THIS TOPIC**

- Biliary complications are a significant concern in liver transplantation, occurring in 15%–25% of cases and often leading to morbidity and mortality. Indocyanine green (ICG) fluorescence has been used in various surgical disciplines for assessing tissue perfusion, but its application in enhancing biliary anastomosis during liver transplantation is not well established and supported primarily by limited case reports.

**WHAT THIS STUDY ADDS**

- This study demonstrates that ICG fluorescence can effectively guide the assessment of bile duct vascularization during liver transplantation. The technique allows for real-time, objective evaluation, enabling precise adjustments at the anastomosis site to potentially reduce biliary complications.

**HOW THIS STUDY MIGHT AFFECT RESEARCH, PRACTICE, OR POLICY**

- The findings support the integration of ICG fluorescence into routine liver transplantation procedures to enhance the safety and precision of biliary anastomosis. This practice could lead to better patient outcomes by minimizing biliary complications, thereby informing future research and potentially influencing surgical guidelines and policies in hepatobiliary surgery. This study constitutes an IDEAL Stage 1 or ‘first in human’ investigation designed to evaluate the use of fluorescence in biliary anastomoses.

## Introduction

 Liver transplantation (LT) is a well-established treatment for end-stage liver disease, acute liver failure, and liver cancer.^1^ Advances in surgical techniques, postoperative care, immunosuppression, and antiviral therapy have significantly improved patient survival, with current 5-year survival rates reported at 70%–75%.^2 3^ Despite these advancements, LT is not a risk-free procedure. Biliary complications are a major source of morbidity in both early and long-term post-transplant periods. The incidence of these complications ranges from 15% to 25%, with an associated mortality rate of approximately 10%.^4 5^ As a result, biliary complications are a significant concern in transplant patients and are often referred to as the ‘Achilles heel’ of LT, frequently necessitating repeated endoscopic and percutaneous biliary interventions and sometimes leading to re-transplantation.

In recent years, several factors contributing to the occurrence of biliary complications post-LT have been identified. Among the most critical factors influencing the success of biliary anastomosis are the adequacy of surgical technique and the prevention of tissue ischemia.^6^

Indocyanine green (ICG) is a safe molecule, approved by the Food and Drug Administration in 1959.^7^ On intravenous administration, ICG binds to alpha-1 lipoprotein and diffuses throughout all vascularized tissues in the body.^8^,^10^ It is subsequently taken up by hepatocytes and excreted unchanged into the bile.^11^ When exposed to near-infrared light, ICG absorbs light at a wavelength of 800 nm and emits strong fluorescence at 830 nm. This fluorescence can be digitally processed and displayed on a screen, providing precise surgical guidance.^12 13^

Due to these properties, ICG has been shown to enable the evaluation of tissue vascularization.^14 15^ Particularly in assessing the perfusion of anastomosis, ICG has proven to be useful in colorectal anastomoses,^16^,^18^ reducing the risk of anastomotic leaks in patients undergoing colorectal surgery.^19^

Traditionally, the vascular perfusion of donor’s and recipient’s bile ducts in LT is assessed by evaluating the macroscopic appearance and observing back-bleeding from the stumps. This method is inherently subjective, and an objective assessment of vascularization at the site of surgical anastomosis is crucial for preventing anastomotic leaks or strictures. The fact that ICG is excreted in bile, combined with its ability to assess tissue perfusion, makes it a potentially valuable tool for ensuring safe biliary anastomoses in LT.

There is limited evidence from small case series on the role of ICG in facilitating safer biliary anastomosis.^20 21^ In this case series, we present our findings on the use of ICG to enhance the safety of biliary anastomosis during LT. This article aims to provide additional evidence on the various applications of ICG and its significance in liver transplant surgery.

## Methods

Between January 2023 and July 2024, a small case series was conducted at Vall d'Hebron University Hospital (Barcelona, Spain), where ICG fluorescence was used to evaluate the vascularization of the bile duct stump in 10 adult LT procedures. Patient recruitment was carried out by sequentially enrolling transplant candidates at our center. Patients undergoing hepaticojejunostomy or retransplantation were excluded due to potential alterations in ICG fluorescence patterns. Oral informed consent was obtained from all patients, informing them that ICG is a safe molecule routinely used as part of standard clinical practice in liver surgery.

### Liver donor

Liver grafts were retrieved from adult donors, including those after brain death (DBD) and circulatory death (DCD). DCD grafts were recovered using in situ normothermic regional perfusion (NRP), established via postmortem cannulation of the abdominal aorta and inferior vena cava. The NRP circuit was maintained for 90–120 min to assess liver function. In DBD, aortic and portal cannulation through the inferior mesenteric vein was performed. All grafts were perfused with IGL-1 solution, then benched on the back table and placed in preservation solution for subsequent transplantation.

### Liver transplant

All procedures were carried out by the same experienced liver transplant team. The liver transplant was performed using the piggyback technique and portacaval shunt, starting with a side-to-side cavo-caval anastomosis, followed by portal vein anastomosis to re-establish liver flow, and concluding with the arterial anastomosis. Intraoperative flow measurements were taken using the Medistim device to assess blood flow and proper vascularization of the graft. Cold and warm ischemia times were recorded.

Before performing the biliary anastomosis, and once the arterial and venous flows were optimized, the estimated cutting point for both the donor and recipient biliary stumps was defined by the surgeon, based on macroscopic appearance and signs of ischemia.

### ICG infusion and biliary stump assessment

ICG was administered via a central venous line by the anesthesia team as a 3 mg bolus dose. The times for ICG uptake by the liver surface and bile ducts were recorded. Fluorescence was detected using the high-definition Stryker Novadaq Pinpoint-camera system, which was wrapped in sterile plastic and displayed on a screen next to the lead surgeon. The camera, designed specifically for open surgery, allowed the surgeon to adjust its position and settings as needed.

30 s after ICG administration, the perfusion levels of the donor and recipient bile duct stumps were assessed. The proximal vascularized part (fluorescent) of the graft bile duct was identified, and the distal non-vascularized part (non-fluorescent) was marked and resected. In the recipient stump, the non-vascularized part (non-fluorescent) was also marked and resected. Stump bile duct bleeding was considered a predictor of good vascularization. Additionally, graft perfusion was assessed during the procedure.

### Biliary anastomosis

Biliary anastomosis was performed following the standardized technique established by Dr Margarit in 1986 at Vall d'Hebron University Hospital. Initially, the bile duct is tutored by placing a 5 or 6 Fr Argyle transcystic tube. A duct-to-duct end-to-end anastomosis is then performed using interrupted monofilament absorbable sutures of 6/0 polydioxanone suture (PDS). The procedure begins with the placement of two reference sutures on the mid-posterior side and one on the mid-anterior side, to approximate the edges and divide the anastomosis into two halves, ensuring optimal exposure. Half of the anastomosis circumference is completed, followed by a 180° flip of the anterior and posterior stay sutures to conveniently complete the remaining half. Finally, saline solution is infused through the Argyle tube to check for any anastomotic biliary leaks.

T-tubes, non-absorbable prostheses or self-expanding absorbable prostheses were not employed for biliary anastomosis. An ICG angiography was performed again after the anastomosis to evaluate correct vascularization.

### Follow-up

After the procedure, patients were admitted to the intensive care unit for close monitoring, followed by a recovery period in the hospital ward until discharge. Patients were followed up in the outpatient clinic with weekly visits for the first 2 months post-transplant, every 15 days during the third month, and every 3 months thereafter.

## Results

Donor, recipient and transplant characteristics are detailed in table 1. In total, three donors were DBD, and seven were DCD. Most donors were elderly, with 60% being ≥70 years old, and 40% had cardiovascular risk factors. All DCD livers experienced a short functional warm ischemic time (<30 min), with stable liver transaminase levels during NRP remaining below three times the upper normal limit.

**Table 1:** Donor, recipient and transplant characteristics

Case	Recipient characteristics						Donor characteristics				Transplant details						Follow-up			
	Age	BMI	CVD	Indication for transplant	Child-Pugh	MELD	Donor	Age	CVD	Anatomical alterations	Total ischemia time (min)	Functional warm ischemia time (min)	Warm ischemia time (min)	Arterial flow (mL/min)	Portal flow (mL/min)	Additional bile duct resection (mm)	Bile duct complications	Liver function tests	Days hospitalized	Follow-up (mo)
#1	50s	25.1	–	Acute liver failure	–	–	DBD	70s	HBP, DM, atherosclerosis	RHA from SMA	420	-	40	450	1300	No	No	NLF	12	18
#2	70s	28.7	DM, HBP	Cirrhosis+HCC	A	19	DCD	70s	–	No	369	16	30	130	3000	2 mm (graft)	No	NLF	10	17
#3	60s	30.1	DM	Cirrhosis	C	21	DBD	70s	HBP, DM, atherosclerosis	No	190	-	23	90	1300	No	No	NLF	17	16
#4	40s	30.6	–	Cirrhosis	B	20	DCD	50s	–	No	225	21	18	130	1400	No	No	NLF	8	16
#5	60s	33.0	Smoker	Cirrhosis	B	13	DCD	70s	HBP, DM, atherosclerosis	No	250	14	32	300	1800	4 mm (graft)	No	NLF	10	16
#6	70s	26.1	Atherosclerosis	Cirrhosis+HCC	A	19	DCD	70s	atherosclerosis	No	187	12	35	170	1000	4 mm (graft)	No	NLF	20	6
#7	60s	21.6	–	Cirrhosis	A	18	DBD	50s	–	No	270	-	35	240	1400	No	No	NLF	10	6
#8	60s	31.2	DM	Cirrhosis	B	13	DCD	70s	–	No	362	18	22	120	1100	2 mm (graft), 2 mm (recipient)	No	NLF	10	6
#9	60s	24.7	Smoker	Cirrhosis+HCC	A	19	DCD	50s	–	No	312	21	27	490	1200	2 mm (graft)	No	NLF	8	6
#10	60s	21.1	DM	Cirrhosis+HCC	A	19	DCD	40s	–	No	290	0 (premortem)	25	150	2300	No	No	NLF	13	6

BMI, body mass index; CVD, cardiovascular disease; DBD, donor after brain death; DCD, donor after circulatory death; DM, diabetes mellitus; HBP, high blood pressure; HCC, hepatocellular carcinoma; MELD, Model for End-Stage Liver Disease; NLF, normalized liver function; RHA, right hepatic artery; SMA, superior mesenteric artery.

The majority of liver grafts were transplanted into middle-aged males (80%). Among the recipients, one had acute liver failure due to hepatitis B virus, five had cirrhosis (from alcohol, hepatitis C virus, and metabolic dysfunction-associated steatotic liver disease), and four had cirrhosis with concomitant hepatocellular carcinoma. Five of the cirrhotic patients were classified as Child-Pugh type A, three as type B, and one as type C. The mean Model for End-Stage Liver Disease score among recipients was 17.8 (range 13–21).

**Figure 1:**
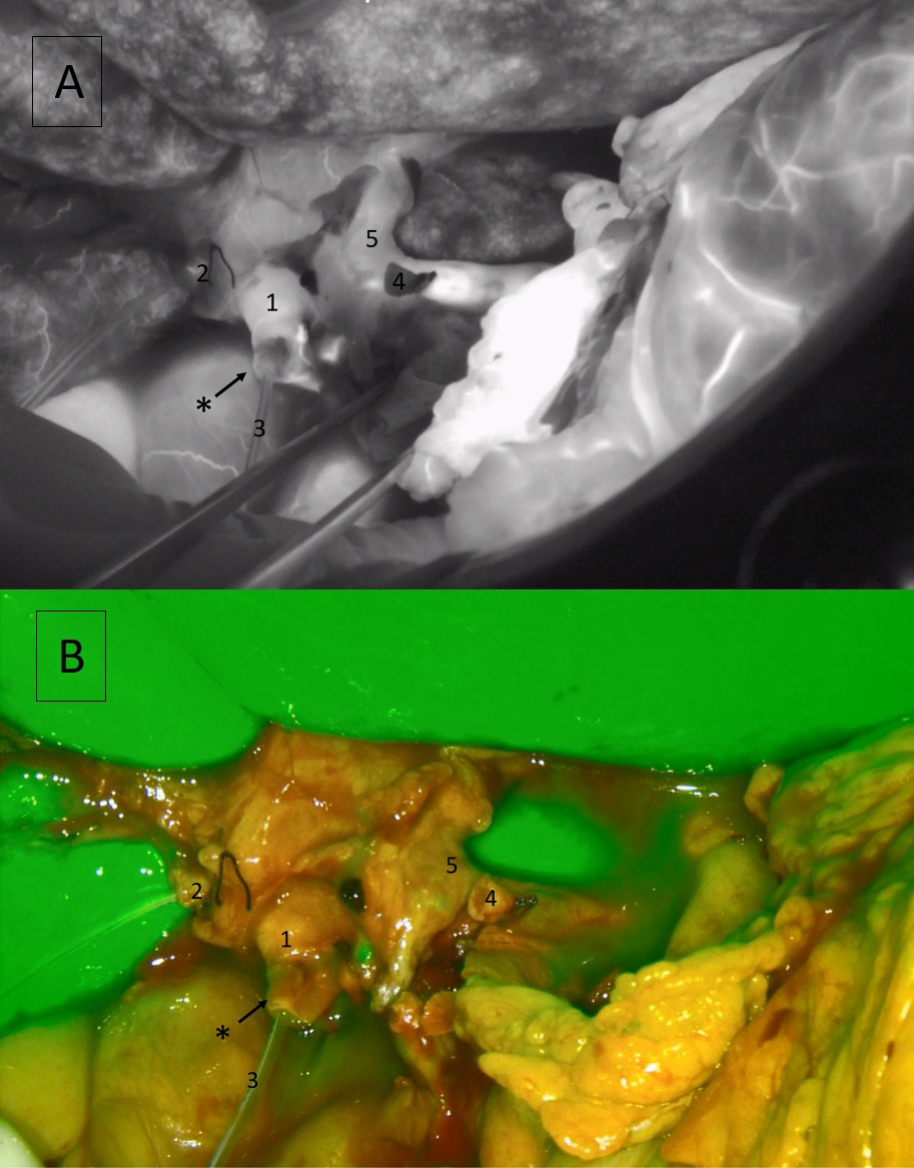
Distal ischemia of the graft bile duct stump. Near-Infrared mode (**A**) and overlay mode (**B**), with a hypoperfused area (non-fluorescent area) (* pointing arrow). 1: common bile duct; 2: cystic stump; 3: Argyle tube; 4: gastroduodenal artery stump; 5: proper hepatic artery.

**Figure 2:**
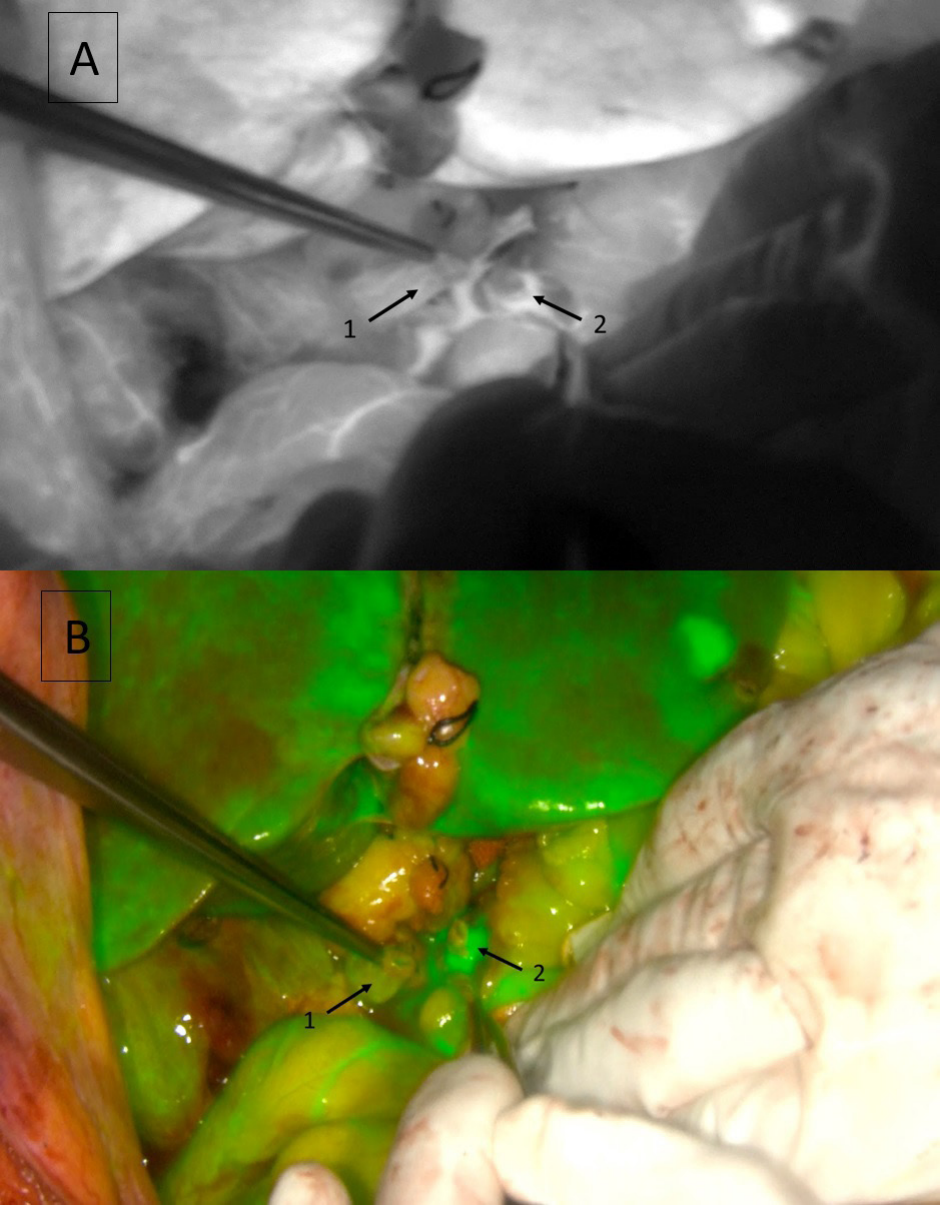
Correct vascularization of graft and receptor bile duct stumps. Near-Infrared mode (**A**) and overlay mode (**B**). 1: graft bile duct stump; 2: receptor bile duct stump.

During liver transplant, the mean flow, once optimized, of the hepatic artery was 227 mL/min (range 90–450 mL/min), and the mean flow of the portal vein was 1580 mL/min (range 1000–3000 mL/min). The ICG fluorescence-guiding technique was successfully applied in all cases. The surgeon initially selected the optimal division point for both bile duct stumps based on the macroscopic appearance and back-bleeding of the graft and the recipient’s bile duct. After ICG administration and assessment, the surgeon adjusted the anastomosis location based on the identification of non-fluorescent areas in five patients (50%) (figure 1). Among these, four were DCD (57% of DCD) and one was a DBD (33% of DBD). In all cases where the anastomosis location was modified (five cases), additional bile duct resection was required in the graft (mean 3 mm), except in one instance where both the graft and the recipient’s bile duct stump (2 mm and 2 mm) required trimming following ICG uptake analysis. For the remaining grafts, the ICG fluorescence findings matched the initial assessment, and no additional resection was performed (figure 2). The use of fluorescence prolonged the operative time by 3–5 min. Videos of the ICG infusion and perfusion were recorded and are attached in the Supplementary Material (onlinesupplemental materials 1  2). No changes in the surgical technique for biliary anastomosis were necessitated after ICG evaluation. No adverse events related to the infusion of ICG occurred during the perioperative or postoperative periods.

The average total ischemia time was 262 min, with an average functional warm ischemia time in DCD (excluding one donor with premortem cannulation) of 17 min and a mean warm ischemia time of 28.7 min. All patients experienced satisfactory short-term outcomes and were discharged after 10–17 days. During long-term follow-up (five patients monitored for >12 months), no biliary complications were reported over an average of 16.6 months, with graft and patient survival at 100%.

## Discussion

Biliary complications remain a major challenge in the postoperative management of LT, despite advancements in surgical techniques and donor management. This study aimed to evaluate the effectiveness of ICG fluorescence in improving the safety of biliary anastomosis during LT. Our findings demonstrate that ICG fluorescence can enhance the precision of biliary anastomosis by providing real-time, objective assessment of bile duct vascularization. This technique successfully guided surgical adjustments in five cases, complementing traditional macroscopic evaluation.

Our results are consistent with the limited literature on the use of ICG for biliary anastomoses. Previous studies have highlighted the potential benefits of ICG in assessing bile duct vascularization. Coubeau *et al*^20^ reported the first case in the use of ICG to identify poorly vascularized areas of the bile duct, leading to modifications in the anastomosis site. Their findings were corroborated by Panaro *et al*,^21^ who demonstrated that ICG-guided resection of hypoperfused bile duct segments could prevent subsequent biliary complications. While these studies were limited by small sample sizes, they suggest that ICG may reduce the risk of biliary complications.

In our study, the use of ICG led to adjustments at the anastomosis site in a subset of cases, particularly for DCD grafts. This adjustment is significant, given the literature indicating that DCD livers are more prone to biliary complications due to prolonged warm ischemia.^22 23^ The ability of ICG to highlight areas of poor perfusion that might otherwise be missed underscores its potential value in managing such high-risk grafts.

The clinical application of ICG fluorescence in LT adds a valuable dimension to the assessment of bile duct vascularization. The technique’s ability to provide real-time, objective feedback complements traditional macroscopic assessments and could potentially reduce the incidence of biliary complications. This aligns with the successful use of ICG in other surgical fields, such as colorectal surgery, where it has been shown to decrease anastomotic complications.^19 24^ The extension of ICG use into hepatobiliary surgery demonstrates its versatility and potential for improving surgical outcomes.

This article introduces an additional application of ICG in hepatobiliary surgery, where it has already shown evidence in reducing bile duct injuries after cholecystectomy^25^,^27^ and in enhancing visualization of extrahepatic biliary ducts during surgical dissection.^28 29^ It also has potential utility in liver transplant surgery, aiding in bile duct identification and partitioning^30^ and facilitating the preoperative identification of hepatic tumors.^31^,^34^

This study is limited by its small sample size and relatively short follow-up period. Despite these constraints, the initial results are promising and support the further exploration of ICG in this context. Larger, prospective studies are needed to validate these findings and fully establish the role of ICG in biliary anastomosis during LT. Additionally, research should focus on evaluating the technique’s impact on different donor types, particularly on DCD or those with significant comorbidities or cardiovascular risk factors, who may benefit the most from improved vascular assessment.

In conclusion, the integration of ICG fluorescence in LT appears to enhance surgical precision and potentially reduce biliary complications by providing a real-time, objective assessment of bile duct vascularization. While the technique is safe and minimally time-consuming, further research is required to confirm its benefits and establish its role in routine clinical practice.

## Supplementary material

10.1136/bmjsit-2024-000322 online supplemental file 1

10.1136/bmjsit-2024-000322 online supplemental file 2

10.1136/bmjsit-2024-000322 Abstract translation 1 This web-only file has been produced by the BMJ Publishing Group from an electronic file supplied by the author(s) and has not been edited for content.

## Data Availability

Data are available upon reasonable request.
