# Supplementary material for: Brightening the path to safe liver transplants: the role of ICG fluorescence in biliary anastomosis
Source: BMJ Surg Interv Health Technol. 2025 Jun 9;7(1):e000322. doi: 10.1136/bmjsit-2024-000322 (PMC12163333; doi:10.1136/bmjsit-2024-000322)
Supplement: Abstract translation 1 [file bmjsit-7-1-s003.docx]

**ABSTRACT (SPANISH)**

**Objetivos**: Evaluar la efectividad de la fluorescencia con verde de indocianina (ICG) en mejorar la seguridad y precisión de la anastomosis biliar durante el trasplante hepático (TH). La hipótesis principal de investigación fue si el ICG podría proporcionar una evaluación objetiva en tiempo real de la vascularización de la vía biliar para reducir las complicaciones biliares postoperatorias.

**Diseño**: Serie de casos prospectiva y observacional.

**Lugar**: Hospital universitario académico de atención terciaria en Barcelona, España.

**Participantes**: Diez pacientes adultos que se sometieron a TH entre enero de 2023 y julio de 2024. Los pacientes fueron seleccionados según la indicación para el TH, con etiologías variadas de insuficiencia hepática. Los donantes incluyeron a aquellos con muerte cerebral (DBD) y asistolia controlada (DCD).

**Intervenciones**: Se administró ICG por vía intravenosa en bolus de 3 mg para evaluar la vascularización de los muñones de la vía biliar. La fluorescencia se visualizó utilizando un sistema de cámaras de alta definición durante la cirugía, y se realizaron ajustes en el sitio de la anastomosis según los patrones de fluorescencia observados.

**Principales Medidas de Resultado**: El resultado principal fue la identificación de tejido biliar no vascularizado (no fluorescente) y los ajustes subsecuentes en el sitio de la anastomosis. Los resultados secundarios incluyeron la incidencia de complicaciones biliares y la supervivencia del paciente durante el período de seguimiento.

**Resultados**: La fluorescencia con ICG identificó con éxito áreas no fluorescentes en los muñones del conducto biliar, lo que llevó a ajustes quirúrgicos en 5 casos (50%), particularmente en injertos de DCD. El procedimiento fue bien tolerado sin eventos adversos relacionados con la administración de ICG. El uso de fluorescencia con ICG añadió un promedio de 3-5 minutos al tiempo operatorio. No se reportaron complicaciones biliares durante el seguimiento, y la supervivencia de los pacientes fue del 100%.

**Conclusiones**: La fluorescencia con ICG proporciona una herramienta objetiva y valiosa para evaluar la vascularización de la vía biliar durante el TH, lo que potencialmente reduce las complicaciones biliares. La integración de esta técnica en la práctica clínica podría mejorar la precisión quirúrgica y los resultados para los pacientes. Se necesita más investigación para confirmar estos hallazgos en poblaciones mayores y diversas.
